# High Confidence Fission Yeast SUMO Conjugates Identified by Tandem Denaturing Affinity Purification

**DOI:** 10.1038/srep14389

**Published:** 2015-09-25

**Authors:** Minghua Nie, Ajay A. Vashisht, James A. Wohlschlegel, Michael N. Boddy

**Affiliations:** 1 Department of Cell and Molecular Biology, The Scripps Research Institute, La Jolla, CA 92037, USA; 2 Department of Biological Chemistry, David Geffen School of Medicine, University of California, Los Angeles, CA 90095, USA

## Abstract

Covalent attachment of the small ubiquitin-like modifier (SUMO) to key targets in the proteome critically regulates the evolutionarily conserved processes of cell cycle control, transcription, DNA replication and maintenance of genome stability. The proteome-wide identification of SUMO conjugates in budding yeast has been invaluable in helping to define roles of SUMO in these processes. Like budding yeast, fission yeast is an important and popular model organism; however, the fission yeast *Schizosaccharomyces pombe* community currently lacks proteome-wide knowledge of SUMO pathway targets. To begin to address this deficiency, we adapted and used a highly stringent Tandem Denaturing Affinity Purification (TDAP) method, coupled with mass spectrometry, to identify fission yeast SUMO conjugates. Comparison of our data with that compiled in budding yeast reveals conservation of SUMO target enrichment in nuclear and chromatin-associated processes. Moreover, the SUMO “cloud” phenomenon, whereby multiple components of a single protein complex are SUMOylated, is also conserved. Overall, SUMO TDAP provides both a key resource of high confidence SUMO-modified target proteins in fission yeast, and a robust method for future analyses of SUMO function.

SUMO (small ubiquitin-like modifier), also called Pmt3 in fission yeast (*Schizosaccharomyces pombe*), is an ~10 kDa protein that shares the beta-grasp structural fold of ubiquitin, despite extreme divergence in their primary sequences. Like ubiquitin, SUMO is conjugated to lysine residues of target proteins to regulate most aspects of cell growth, including cell cycle transitions and genome stability^1^,^2^,^3^. SUMO conjugation requires a cascade of E1 activating (e.g. UBA2^SpFub2^-SAE1^SpRad31^), E2 conjugating (Ubc9) and E3 ligase factors (e.g. PIAS1^SpPli1^), in which E3 ligases enhance the efficiency and specificity of target SUMOylation^3^. There are more than a thousand ubiquitin E3 ligases, but far fewer (<10) known SUMO E3 ligases. The main SUMO E3 ligases are the protein inhibitor of activated STAT (PIAS) family, which has several members in mammalian cells e.g. PIAS1-4, two in budding yeast SIZ1/2 and one called Pli1 in fission yeast^4^,^5^. Pli1 is responsible for >90% of SUMOylation in fission yeast, as detected by western analysis^6^.

SUMO affects the function of its targets in many ways, including driving new protein-protein interactions, altering their subcellular localization, or flagging them for proteasomal degradation^1^,^2^,^3^,^7^,^8^. SUMO signals are decoded by specific “reader” or receptor motifs in the proteome that non-covalently bind SUMO, called SUMO interacting motifs (SIMs;^9^,^10^). Tandem arrangements of these motifs can support the recognition of SUMO chains. Furthermore, the presence of both SIMs and ubiquitin interacting motifs (UIMs) in a protein allows the protein to simultaneously recognize SUMO and ubiquitin, thereby increasing selectivity in signaling through these posttranslational modifiers. Compound SUMO and ubiquitin signals can be generated by the SUMO-targeted E3 ubiquitin ligase (STUbL), which ubiquitinates SUMOylated proteins^8^,^11^. One “reader” of such hybrid signals is the Cdc48 (p97) AAA+ATPase cofactor Ufd1, which contains both UIM and SIM motifs, and promotes the chromatin extraction and/or proteasomal degradation of STUbL target proteins^12^,^13^,^14^. Another, RAP80, is a key BRCA1 cofactor in DNA repair and is recruited to DNA lesions via UIM and SIM-dependent recognition of SUMO-ubiquitin hybrid chains formed by the action of the human STUbL RNF4^15^.

SUMO conjugation is a highly reversible process, as Ubl-specific proteases (ULPs) can efficiently remove SUMO from its targets^16^,^17^. In yeast there are two ULPs, Ulp1 and Ulp2, which each desumoylates a subset of SUMO conjugates, with selectivity arising largely from their distinct subcellular localizations. Ulp1 (HuSENP1/2) localizes to nuclear pores, whereas Ulp2 (HuSENP6/7) is nucleoplasmic^16^,^18^. These desumoylating activities are highly active upon cell lysis, and together with the generally low stoichiometry of SUMO modification, make SUMO target identification challenging. To help circumvent these issues, we used Tandem Denaturing Affinity Purification (TDAP) of SUMO coupled with mass spectrometry to identify SUMO conjugates proteome-wide in fission yeast.

## Results and Discussion

### Implementation of TDAP in fission yeast (*Schizosaccharomyces pombe*)

**Figure 1:**
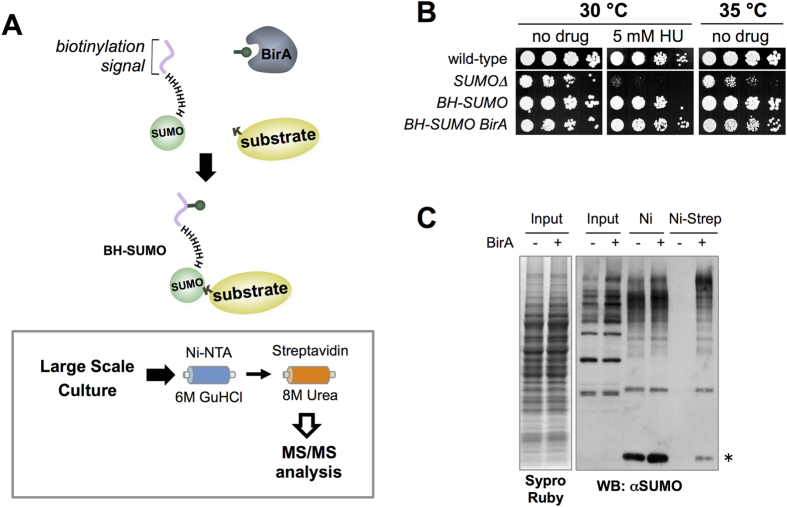
Tandem Denaturing Affinity Purification (TDAP) of SUMO targets. (**A**) Schematics of TDAP. See the main text for details of the method. (**B**) Spot assay of the indicated strains on YES plates, with or without hydroxyurea (HU). (**C**) BH-SUMO expressed from endogenous locus in strains with (+) or without (−) pAdh-driven BirA expression. SUMO conjugates were sequentially purified on Ni-NTA and streptavidin beads. Input and eluate after a single step (Ni) or tandem (Ni-Strep) purification were analyzed by Western blotting with a SUMO antibody. The asterisk marks the position of free BH-SUMO. Sypro Ruby staining of the input is shown on the left as a loading control.

To identify fission yeast SUMO conjugates, we adapted a method that was previously used to purify unstable ubiquitin conjugates from budding yeast^19^. A module containing a minimal biotinylation site^20^ and a hexahistidine (H_6_) tag were added to the amino terminus of endogenous SUMO (BH-SUMO). This combination of epitopes enables tandem affinity purification of target proteins under strongly denaturing conditions (TDAP) (Fig. 1A). Such conditions inactivate desumoylating enzymes upon cell lysis, thereby protecting the otherwise highly labile SUMO conjugates. Moreover, the stringent purification afforded by TDAP yields high confidence SUMO conjugates.

To ensure efficient biotinylation of BH-SUMO, *E. coli* BirA was constitutively co-expressed from the Adh promoter. BH-SUMO is functional, and overexpression of BirA caused only a minor reduction in growth rate and no increase in sensitivity to genotoxins or elevated temperature, as compared to SUMO delete cells (Fig. 1B). TDAP of BH-SUMO gave the expected results, as SUMO conjugates were captured on nickel columns independently of BirA expression, but their binding to streptavidin columns was BirA-dependent (Fig. 1C).

Having established the stringent TDAP strategy, we executed duplicate large-scale BH-SUMO purifications (see Methods for details) on strains NBY2691 and NBY2636 (Table 1). The experimental strain NBY2691 expresses both BH-SUMO and BirA, whereas the control strain NBY2636 expresses BH-SUMO but not BirA (Table 1, Fig. 1C). Data from mass spectrometric analyses of duplicate TDAPs were combined for each of the NBY2691 and NBY2636 strains. Out of a total of 195 proteins identified by mass spectrometry, 166 were candidate SUMO conjugates, as they were not found in the control (see Supplementary Table S1, S2). Amongst the 29 background proteins were the abundant and endogenously biotinylated metabolic enzymes Pyr1 and Cut6. These factors were carried through the initial nickel affinity purification, and due to their essential functions cannot be deleted to genetically eliminate these as contaminants. Other contaminants include the abundant copper amine oxidases Cao1 and Cao2, and a protein of unknown function SPCC594.01. These proteins all contain intrinsic poly-histidine tracts that allow them to bind the Ni-NTA resin. Likely as a result of their abundance, they persist through the secondary purification on streptavidin.

There are currently no other global SUMO proteomic studies in fission yeast with which to compare our data. However, in comparison to earlier studies in budding yeast^10^,^21^,^22^,^23^,^24^, 166 candidate SUMO targets is an average number.

### Direct validation of select SUMO conjugates identified by TDAP

The abundance of the SUMO-modified form of a given protein is very low (generally <1%) compared to that of the unmodified form. Therefore, various cellular stresses including genotoxins and heat shock, or SUMO protease deletions, are used to increase the SUMO-modified pool of proteins and aid in their detection e.g.^10^,^23^. Here we used heat stress that promotes a general increase in SUMO conjugation to both heat shock factors and proteins not specific to the heat stress response (see below).

**Figure 2:**
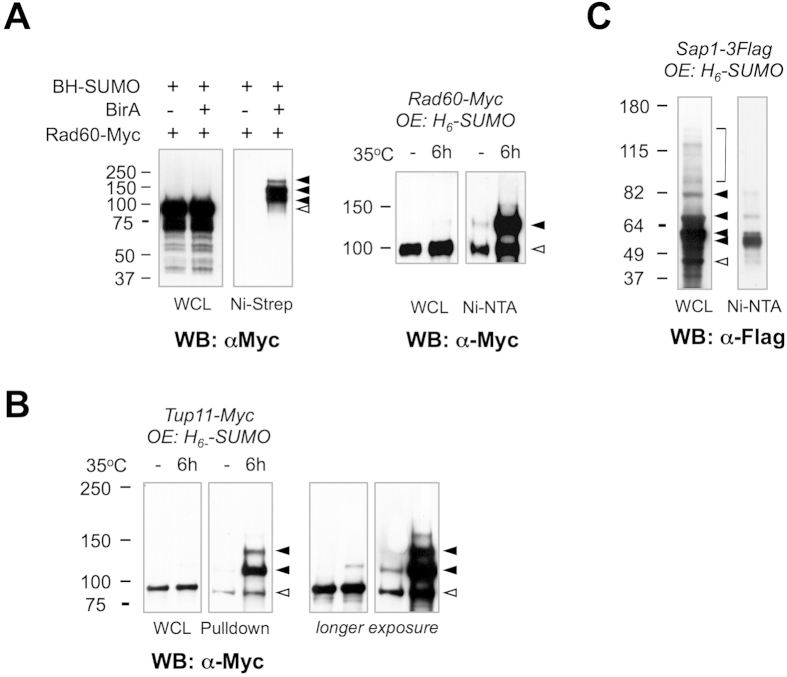
Validation of SUMO targets identified by mass spectrometry. (**A**) *Left panel*: Rad60-Myc and BH-SUMO were expressed from endogenous loci in strains with (+) or without (−) BirA expression. The whole cell lysate (WCL) and eluate after TDAP (Ni-Strep) were analyzed by Western blotting with a Myc antibody. *Right panel*: A single-step Ni-NTA pulldown was performed using a Rad60-Myc strain that overexpressed (OE) 6-his-tagged SUMO (H_6_-SUMO) grown at 25 °C, or shifted to 35 °C for 6 h. The WCL and eluate from Ni pulldown (Ni-NTA) were analyzed by anti-Myc Western blotting. (**B**) Ni-NTA pulldown was performed using an endogenously tagged Tup11-Myc strain overexpressing (OE) H_6_-SUMO grown at 25 °C, or shifted to 35 °C for 6 h. The WCL and eluate after Ni pulldown (Ni-NTA) were analyzed by anti-Myc Western blotting. A longer exposure is shown on the right. (**C**) Ni-NTA pulldown was performed on an endogenously tagged Sap1-3xFlag strain overexpressing (OE) H_6_-SUMO grown at 30 °C. The WCL and eluate after Ni-NTA pulldown were analyzed by anti-Flag Western blotting. In all panels, open triangles denote the position of unmodified protein species, while solid triangles mark the positions of SUMO-modified species. For the pulldown assays, the protein lysate was quantitated by OD_280_ and equivalent amount of total proteins was used in conditions indicated in the figure.

To confirm SUMOylation of several of our candidate SUMO conjugates we epitope tagged the DNA repair protein Rad60^25^,^26^, the transcriptional corepressor Tup11^27^, and the essential chromatin factor Sap1^28^ at their endogenous loci. Repeating the TDAP purification *in the absence of heat stress* resulted in the highly specific enrichment of SUMO conjugated Rad60 (Fig. 2A, left panel). Therefore, heat stress enriches not only for heat stress-specific factors, but also more generally for low abundance SUMO conjugates. The SUMOylated form of Rad60 could also be detected following single step denaturing purification of ectopically expressed 6His-SUMO, and as anticipated the detection of the SUMO-conjugated form of Rad60 was enhanced by heat stress (Fig. 2A, right panel). Notably, some unmodified Rad60 was co-purified, underscoring the utility of TDAP stringency to reduce potential false positives in mass spectrometry (Fig. 2A, right panel).

Rad60 and its budding yeast orthologue ESC2 function as cofactors for the SUMO E3 ligase Nse2, a subunit of the Smc5-Smc6 DNA repair complex^29^,^30^. All components of the SUMO pathway are modified by SUMO, so given its intimate connection with Nse2 the SUMOylation of Rad60 may be anticipated. Whether this modification is regulatory remains to be addressed in the future.

SUMO conjugated forms of both Tup11 and Sap1 were similarly enriched following 6His-SUMO purification in the *presence or absence* of heat stress (Fig. 2B,C). These results again underscore heat stress as a method to enhance SUMO conjugation to factors that are not necessarily specific to the stress response. Tup11 is a homologue of the budding yeast transcriptional corepressor Tup1^27^, whose regulated SUMOylation and deSUMOylation impacts transcription kinetics^31^,^32^,^33^. Therefore, analogous regulation of Tup11 and transcription by SUMO can be anticipated in fission yeast. Sap1 is a DNA binding protein present at various chromosomal loci that acts as a replication fork barrier, in addition to recruiting the replication fork protection complex and playing a role in replication checkpoint activation^34^,^35^,^36^. Given that Sap1 is a “hub” factor in these key DNA replication transactions, it is an attractive candidate mediator of some of the genome stability roles of SUMO.

Although not validated herein, we recently showed that as in other species fission yeast Top1 is a *bona fide* SUMO conjugate^37^. In addition, the translation initiation factor eIF4G was recently independently found to be SUMOylated^38^.

Notably, of the 166 candidate SUMO conjugates in fission yeast, 148 have clear orthologues in budding yeast, 85 (57.4%) of which are known SUMO substrates (Supplementary Table S1). An additional 15 of these budding yeast orthologues interact with SUMO pathway factors, despite a lack of direct evidence for their SUMOylation (Supplementary Table S1). The >50% overlap between our SUMOylation data and that of the compiled budding yeast analyses^24^ provides cross-validation of both datasets. Overall, these data indicate that TDAP enables confident identification of SUMO conjugates, and that our proteomic data will be a useful resource.

### Conservation of SUMO regulated processes and the SUMO “cloud” phenomenon

Gene ontology (GO) analysis of the 166 candidate fission yeast SUMO conjugates revealed overlap with processes controlled by SUMO in budding yeast (Table 2, Supplementary Table S3). For example, chromosome organization, ribosome biogenesis, and macromolecular complex assembly are highly represented in both fission and budding yeasts. This observation is consistent with our current understanding of the biological roles of SUMO in various organisms^1^,^2^,^3^,^39^,^40^.

Transcription factors are major targets of SUMO modification across species^3^,^41^, which our data confirms to also be the case in fission yeast (Supplementary Tables S1 & S3). Moreover, as initially reported in budding yeast^22^,^42^, we note that multiple components of a given complex are SUMOylated (Table 3). For example, 7 members of the TFIID/SAGA complex were identified in our analysis. Multiple components of the SAGA complex are SUMOylated in budding yeast, albeit not the same ones we identified^39^; and human TFIID is SUMOylated on both Taf12 and Taf5^22^,^43^. In addition, we detected SUMOylation of two or more components of several other chromatin associated complexes including RSC, COMPASS, Swr1 and Ino80, which are likewise SUMOylated coordinately in budding yeast^22^. Thus, the apparently promiscuous modification of several proteins within a complex^40^, or SUMO “cloud”, is a conserved feature of SUMO modification in fission yeast.

As with previous approaches, TDAP is not saturating and there are a number of known fission yeast SUMO conjugates not identified in our analysis e.g. the RecQ family helicase Rqh1 (human BLM)^44^, the telomere associated factor Tpz1^45^,^46^, the homologous recombination repair protein Rad52^13^,^47^, the heterochromatin protein (HP1) homologue Swi6^48^ and the DNA repair/chromatin organization factor Smc6^49^. The absence of these factors from our mass spectrometry data likely reflects the standard limitations of all such approaches. One is an issue of protein abundance and stoichiometry of SUMOylation, wherein the most abundant and/or heavily SUMOylated proteins will dominate the mass spectrometry analysis, thereby reducing the ability to detect low abundance SUMO conjugates. In addition, the SUMOylation of certain proteins may be context specific i.e. be stimulated in response to DNA damage. This is the case for Smc6 and Rqh1, whose SUMOylation is strongly stimulated in the presence of the DNA alkylating agent methyl methanesulfonate^44^,^49^. In this regard, the coordinated SUMOylation of functionally related factors in response to certain genotoxic stresses was recently reported^39^. Therefore, applying the now validated TDAP approach in fission yeast that have been exposed to genotoxic stress is expected to yield additional targets within the relevant DNA repair and replication pathways.

Overall, we have implemented and validated a new approach for the highly stringent identification of SUMO conjugates through TDAP and mass spectrometry. This result provides an excellent resource for the analysis of SUMO pathway function in fission yeast, which will also be of interest to the broader community studying the physiological impacts of this critical posttranslational modifier.

## Methods

### General yeast techniques

Standard methods for *S. pombe* were performed as described previously^50^. All strains (Table 1) are of genotype *ura4-D18 leu1-32* unless otherwise stated.

### Plasmid construction

To express *E. coli* BirA in *S. pombe*, pMN017 (pREP42adh-BirA) plasmid was linearized by restriction digest with MluI and integrated at *ars1* on chromosome 1 of *S. pombe*. The construction of pMN017 was achieved in two steps: first, the *S. pombe adh* promoter was PCR amplified from the pART vector^51^ and used to replace the *nmt* promoter in pREP42^52^ at the PstI and SalI sites to create pREP42adh; second, the BirA sequence was PCR amplified and inserted into pREP42adh at the SalI and BamHI sites.

### Spot assays

Cells were grown at 25 °C to logarithmic phase (optical density at 600 nm [OD_600_] of 0.6 to 0.8), spotted in 5-fold dilutions from a starting OD_600_ of 0.5 on YES plates or YES plates supplemented with the relevant drug. The plates were then incubated at 25 to 35 °C for 3 to 5 days.

### Tandem Denaturing Affinity Purification (TDAP) of SUMO conjugates

Six liters of cells grown to exponential phase were heat-shocked at 42 °C for 1 h before harvesting by centrifugation. The cell pellet was re-suspended in 120 ml of Lysis Buffer (6 M GuHCl, 50 mM NaH_2_PO_4_, 50 mM Tris.HCl, pH 8.0, and 8 mM imidazole) supplemented freshly with 20 mM N-Ethylmaleimide, 2 mM PMSF, and 2 Complete Protease Inhibitor cocktail tablets (Roche). The cells were lysed with 0.5 mM glass beads in a Bead-Beater (Biospec) with four 1-min pulses, and 5-min chilling between the pulses. Over 90% cell lysis was achieved as examined under a microscope. The lysate was separated from the glass beads by filtration using a COORSTEK Buchner Ceramic Funnel lined with Whatman filter paper. The lysate was clarified by centrifugation at 28000 *g* for 15 min at 4 °C. Supernatant, containing approximately 0.8 g of protein was batch bound to 4 ml of Ni-NTA beads that were pre-equilibrated with Lysis Buffer in a chromatography column at room temperature (RT) for 2 h. The beads were washed four times with 40 ml of Buffer **A** (8 M urea, 50 mM NaH_2_PO_4_, 50 mM Tris, 300 mM NaCl, pH 8, 1 mM PMSF, and 10 mM imidazole). To elute protein from the Ni-NTA beads, 5 ml of Buffer **A** at pH 4.3 was added, and the pH was adjusted to 4 by directly adding 1 M HCl dropwise. The pH of eluted protein solution was immediately adjusted back to 8 with 1 M NaOH. The eluate was bound to 180 μl of streptavidin beads overnight at RT. The beads were washed sequentially with 4.5 ml each of Buffer **B** (8 M urea, 2% SDS, 100 mM Tris, 200 mM NaCl, pH 8), **C** (8 M urea, 0.2% SDS, 100 mM Tris, 1.2 M NaCl, 10% EtOH, 10% 2-propanol, pH 8), **D** (8 M urea, 0.2% SDS, 100 mM Tris, 200 mM NaCl, 10% EtOH, 10% 2-propanol, pH 5), **D’** (8 M urea, 0.2% SDS, 100 mM Tris, 200 mM NaCl, 10% EtOH, 10% 2-propanol, pH 9), and **E** (8 M urea, 100 mM Tris, pH 8). Proteins used for mass spectrometry analysis were digested by trypsin directly on beads. For Western blotting the proteins were eluted with 2 bed-volumes of 2x SDS loading buffer (100 mM Tris, pH 6.8, 200 mM dithiothreitol, 4% SDS, 0.2% bromophenol blue, 20% glycerol) at 95 °C for 5 min.

### Shotgun proteomic analysis of SUMO conjugates

SUMO conjugates isolated using tandem denaturing affinity purification were processed directly from beads for downstream MS analysis. Briefly, the beads were mixed with digestion buffer (100 mM Tris-HCl, pH 8.5, 8 M urea), reduced and alkylated by sequential treatment with 5 mM tris(2-carboxyethyl) phosphine (TCEP) and 10 mM iodoacetamide as described earlier^53^,^54^. Samples were digested with sequential addition of endopeptidase Lys-C and trypsin as described^54^. The peptide mixture was desalted on line and analyzed by 2D-LC-MS/MS on a Thermofisher LTQ-Orbitrap XL Hybrid Ion Trap-Orbitrap Mass Spectrometer as described^53^,^54^. Database searching of the MS/MS spectra was performed using the ProLuCID algorithm^55^. For confident protein and peptide identifications, the results were filtered using DTASelect^56^,^57^ and required at least two unique peptides per protein and a peptide-level false detection rate of less than 5% as estimated by a decoy database strategy^58^. These filtering criteria result in a protein-level FDR of less than 1% for each run. Normalized spectral abundance factor (NSAF) values were calculated as described^59^ and multiplied by a factor 10^5^ for ease of readability. All samples were analyzed in duplicate with complete peptide and proteins lists included in the supplemental material.

### Western Blotting

Western blotting was carried out as previously described^14^. The membrane was probed with antibodies against FLAG (M2, Sigma-Aldrich), Myc (9E10, Covance), or *S. pombe* SUMO^30^, and an HRP-conjugated secondary antibody (Pierce). HRP was detected using the ECL Dura system (Pierce).

### Gene Ontology (GO) analysis

Gene Ontology (GO) enrichment analysis was done using the Princeton implementation of GO term finder (http://go.princeton.edu/cgi-bin/GOTermFinder)^60^. The *p*-values were calculated using a hypergeometric test, and cut-off was set at 0.01 (Table 2, S3).

## Additional Information

**How to cite this article**: Nie, M. *et al.* High Confidence Fission Yeast SUMO Conjugates Identified by Tandem Denaturing Affinity Purification. *Sci. Rep.* **5**, 14389; doi: 10.1038/srep14389 (2015).

## Supplementary Material

Supplementary Dataset 1

Supplementary Dataset 2

Supplementary Dataset 3

## Figures and Tables

**Table 1:** List of yeast strains used in this study.

Strain	Genotype
NBY780	*h*^*+*^
NBY1457	*SUMO::ura4*^*+*^
NBY2636	*BH*-*SUMO*
NBY2691	*BH*-*SUMO* pREP42(*adh*)-BirA*:ura4* integrated at *ars1*
NBY3056	*rad60*-*myc*_*13*_*:kanMx6 BH*-*SUMO*
NBY3057	*rad60*-*myc*_*13*_*:kanMx6 BH*-*SUMO* pREP(*adh*)-BirA*:ura4* integrated at *ars1*
NBY3350	*rad60*-*myc*_*13*_*:kanMx6* pREP41-H_6_-*SUMO*_*GG*_*:LEU2*
NBY3366	*sap1*-*FLAG*_*3*_*:kanMx6* pREP41-H_6_-*SUMO*_*GG*_*:LEU2*
NBY3690	*tup11*-*myc*_*13*_*:kanMx6* pREP41-H_6_-*SUMO*_*GG*_*:LEU2*

**Table 2:** Comparison of top Gene Ontology (GO) categories of *S. cerevisiae* and *S. pombe* SUMO conjugates.

Description	GO ID	*S. pombe*			*S. cerevisiae*		
		x	n	*p*-*value*	x	n	*p*-*value*
chromatin organization	6325	37	242	4.75E-14	91	278	7.24E-33
regulation of transcription	6355	40	410	1.12E-08	150	709	1.36E-31
macromolecular complex subunit organization	43933	47	571	5.49E-08	173	895	4.03E-32
RNA biosynthetic process	32774	43	490	5.62E-08	172	773	1.31E-40
ribosome biogenesis	42254	31	338	1.42E-05	73	444	1.62E-07

x = number of hits amongst SUMO conjugates, n = total number of proteins in the proteome for a specific GO category.

**Table 3:** Group SUMOylation of protein complexes.

Protein Complex	SUMOylated Subunits
TFIID/SAGA	Taf4, Taf9, Taf10, Taf12, Taf111, Spt20, Ngg1
RSC	Rsc1, Rsc4, Ssr1, Snf21
INO80	Ino80, Ies2
Clr6	Clr6, Cph2, Cti6
Tup1	Tup11, Tup12, Ssn6
Swr1	Swr1, Swc4, Msc1
Set1c/COMPASS	Set1, Spf1
TFIIIB	bdp1, brf1
